# Establishing prevalence of diagnosis of personality disorder across high secure forensic services using the ICD 10 and ICD 11 classification

**DOI:** 10.1192/bjo.2021.773

**Published:** 2021-06-18

**Authors:** Anju Soni, Samrat Sengupta, Ian Treasaden

**Affiliations:** 1 South London and Maudsley NHS trust and Broadmoor Hospital; 2 Broadmoor Hospital; 3 West London NHS Trust

## Abstract

**Aims:**

There has been an increasing recognition of the lack of clinical validity of different types of ICD10 personality disorder.

The prevalence was established among patients in a high secure hospital in England of those with either a primary or secondary diagnosis of personality disorder and its recorded type according to ICD10 and then ICD11.

The new ICD11 classification increased the validity of diagnosis of personality disorder as well as its severity.

**Background:**

ICD 11 has proposed the dropping of the classification of personality disorder based on particular types of personality disorder and instead adopting a diathesis model based on 2 dimensions: presence of personality disorder and three levels of severity (Mild, Moderate and Severe) and the option of specifying one or more prominent trait domain qualifiers (Negative Affectivity, Detachment, Disinhibition, Dissociality, and Anankastia) and also specify a Borderline Pattern qualifier.

**Method:**

The electronic medical records were used to establish the presence and type of personality disorder using the criteria of ICD10 and ICD11.

The researchers assured reliability by rating some vignettes using the Schedule for Personality Assessment from Notes and Documents (SPAN-DOC) before rating actual cases.

**Result:**

From a total population of 208 patients, 64(30.8%) were classified as having either a primary or secondary diagnosis of personality disorder according to the ICD 10.

30 (47%) had dissocial personality disorder (DSPD), 19(30%) emotionally unstable personality disorder (EUPD) and 8(13%) paranoid personality disorder. 20 (31%) had a comorbid diagnosis of mental illness and about a tenth had diagnoses of multiple personality disorders. These types of personality disorder diagnosed by the researchers using ICD 10 did not always match the types of personality disorder diagnosed by clinicians at the hospital.

All patients met the criteria of personality disorder under ICD 11 but the number with a borderline specifier was greater than those with an ICD10 diagnosis of EUPD. Using the trait domain qualifiers in ICD 11, patients with ICD 10 diagnoses of EUPD or DSPD showed dissociation and disinhibition, with those with a DSPD showing low and those with EUPD high negative affectivity.

**Conclusion:**

The results confirm that while psychiatrists in a high secure hospital reliably diagnose the presence of a personality disorder, they are much less able to make an accurate diagnosis as to the actual type of personality disorder. The new ICD 11 classification will increase the clinical validity of the diagnosis of personality disorder and its severity.

